# Will Residency Program Directors Look at My United States Medical Licensing Examination (USMLE) Step 1 Score During the 2022-2023 Application Cycle? A National Survey of Program Directors

**DOI:** 10.7759/cureus.29483

**Published:** 2022-09-23

**Authors:** Michael S Powell, Quentin E Parker, Laila L Rhodes, Sagar T Mehta

**Affiliations:** 1 Medicine, University of Arkansas for Medical Sciences, College of Medicine, Little Rock, USA; 2 Health Behavior and Health Promotion, The Ohio State University, College of Public Health, Columbus, USA; 3 Plastic and Reconstructive Surgery, University of Arkansas for Medical Sciences, Little Rock, USA

**Keywords:** national survey, residency program director, residency application, usmle step 1 pass/fail, step 1 pass/fail

## Abstract

Background

The 2022-2023 residency match cycle will be the first cycle that program directors will have to consider some applicants with a numerical United States Medical Licensing Examination (USMLE) Step 1 score while other applicants will only report pass/fail for USMLE Step 1. Previous studies have explored how USMLE Step 1 becoming pass/fail will alter the residency selection process, but it is not yet known when program directors from each specialty expect those changes to be implemented.

Methods

Residency program director’s contact information was extracted from the American Medical Association (AMA) residency program site, Fellowship and Residency Electronic Interactive Database (FREIDA). Of the 5190 programs, 4877 were determined eligible for this study of which 1274 (26.8%) responded.

Results

Of the 1274 US residency program directors included in this survey, 77.0% do not intend to adjust their usage of USMLE Step 1 as a metric in candidate evaluation until the score is no longer reported.

Conclusion

Residency candidates applying during the upcoming cycle can expect the majority of residency programs will not significantly alter their previous utilization of an applicant’s USMLE Step 1 score during the current 2022-2023 residency match cycle.

## Introduction

For years, the United States Medical Licensing Examination (USMLE) Step 1 numerical score has been a tool utilized by residency program directors to assess applicants for interview and selection into their programs [1-3]. As of January 26, 2022, all USMLE Step 1 examinations are being reported as pass/fail alone, while all USMLE Step 1 examinations completed before this date were reported as both pass/fail and numerical score [4].

Historically, metrics such as USMLE Step 1 scores, Step 2 clinical knowledge (CK) scores, Medical Student Performance Evaluation (MSPE), and grades in clinical clerkships consistently rank high among most specialties [1-3]. In reports since the announcement of USMLE Step 1 becoming pass/fail, residency program directors report they will place an increased emphasis on USMLE Step 2 scores and medical school applicants attended [5-7]. Likewise, many within the medical community believe that USMLE Step 2 scores will be the new objective metric used, in place of USMLE Step 1 [8].

The 2022-2023 residency match represents a unique challenge as the first application cycle that program directors will need to consider applicants with a numerical USMLE Step 1 score alongside a portion of applicants that received only a pass/fail result from their USMLE Step 1. While surveys have been conducted to assess the opinions of residency program directors about the changes to Step 1 scoring and which metrics they expect to emphasize, no studies have been conducted to assess when program directors intended to transition to metrics that do not include an applicant’s USMLE Step 1 score.

This study presented our findings regarding when residency program directors expect they will begin to disregard the usage of the numerical USMLE Step 1 score in their evaluation process. It seeks to provide some clarity for residency applicants as they consider how best to optimize their transition from undergraduate to graduate medical education.

## Materials and methods

A total of 4877 program directors’ email addresses were obtained from the American Medical Association residency program site, Fellowship and Residency Electronic Interactive Database (FREIDA). Of the eligible programs, 118 email addresses were returned as undeliverable and additional five programs requested not to be included in the study. Three subsequent survey invitations were emailed to residency program directors (RPDs) between March 3 and March 9, 2020. Program directors who elected to participate utilized a digital link to the Research Electronic Data Capture (REDcap) survey. After completion of the survey, program directors were temporarily able to view graphs summarizing the survey data. The survey was closed on March 14, 2020, at 12 pm CST.

This study presents RPDs’ responses to the question - when is your program likely to begin disregarding three-digit USMLE Step 1 scores? - that was asked as a component of a broader 14-question online survey of RPDs (figures in Appendices). Survey responses were reviewed and analyzed in SPSS version 27 (Armonk, NY: IBM Corp.). Frequency distribution and descriptive statistics were considered to summarize participant responses as relevant to the objective and further elucidate the characteristics of the programs that participated in the study. The study was approved using exempt procedures by the Institutional Review Board at the University of Arkansas for Medical Sciences prior to dissemination of the survey (IRB approval number: 260779).

## Results

Demographics

​Of the 1274 program directors that responded, 1164 selected their specialty. The specialties with the highest response rate relative to their specialty were - physical medicine and rehabilitation (46.1%), emergency medicine (40.3%), and ENT (33.1%) (Table 1). The type of programs overseen by respondents are - university based (58.0%), community based (24.4%), and hybrid (17.5%).

**Table 1:** Survey response rate by specialty.

Specialty	Surveys completed	% of survey results	% response rate of specialty
Family medicine (673)	128	9.8%	19.0%
Internal medicine (672)	124	9.5%	18.5%
Emergency medicine (253)	102	7.9%	40.3%
Obstetrics and gynecology (275)	80	6.3%	29.1%
Pediatrics (246)	79	6.0%	32.1%
Surgery-general (319)	71	5.3%	22.3%
Radiology-diagnostic (191)	54	4.3%	28.3%
Psychiatry (261)	52	4.0%	19.8%
Neurology (156)	48	3.8%	30.8%
Physical medicine and rehabilitation (89)	41	3.1%	46.1%
ENT (118)	39	3.0%	33.1%
Anesthesiology (152)	38	3.0%	25.0%
Orthopedic surgery (192)	35	3.0%	18.2%
Urology (140)	35	2.6%	25.0%
Pathology-anatomic and clinical (140)	32	2.4%	22.9%
Dermatology (141)	27	2.3%	19.1%
Neurological surgery (113)	25	1.9%	22.1%
Ophthalmology (120)	25	1.9%	20.8%
Radiation oncology (89)	24	1.8%	27.0%
Plastic surgery-integrated (76)	23	1.7%	30.3%
Interventional radiology-integrated (84)	22	1.7%	26.2%
Child neurology (73)	20	1.5%	27.4%
Vascular surgery-integrated (62)	16	1.2%	25.8%
Medical genetics and genomics (48)	13	1.1%	27.1%
Thoracic surgery-integrated (28)	6	0.4%	21.4%
Nuclear medicine (39)	5	0.4%	12.8%
Specialty not selected (N/A)	110	10.1%	-
Total	1274	100.0%	26.8%

In an attempt to better understand when program directors anticipate adjusting their metrics we asked, “when is your program likely to begin disregarding three-digit USMLE Step 1 score?” Of the 1274 respondents, the majority (77%) of program directors across all specialties responded that they do not expect to discontinue using applicants USMLE Step 1 numerical score until it is no longer reported (Table 2).

**Table 2:** Utility of numerical USMLE Step 1 score in the pass/fail era. USMLE: United States Medical Licensing Examination

When is your program likely to begin disregarding three-digit USMLE Step 1 score?	Specialty	2021	2022	2023	We are not likely to make changes to our evaluation process until Step 1 stops releasing three-digit score	USMLE Step 1 is not a primary metric used by our program for selecting applicants
	Anesthesiology (n=38)	2	1	1	30	4
	Child neurology (n=20)	0	1	0	14	5
	Dermatology (n=27)	1	0	0	23	3
	Emergency medicine (n=102)	6	2	0	78	16
	Family medicine (n=128)	16	5	0	64	43
	Internal medicine (n=124)	7	4	2	100	11
	Interventional radiology-integrated (n=22)	1	0	0	20	1
	Medical genetics and genomics (n=13)	0	0	0	10	3
	Neurological surgery (n=25)	1	0	1	22	1
	Neurology (n=48)	1	4	1	39	3
	Nuclear medicine (n=5)	0	0	0	2	3
	Obstetrics and gynecology (n=80)	6	4	1	61	8
	Ophthalmology (n=25)	1	1	0	23	0
	Orthopedic surgery (n=35)	4	1	0	30	0
	Otolaryngology-head and neck surgery (n=39)	1	4	0	30	4
	Pathology-anatomic and clinical (n=32)	0	0	0	28	4
	Pediatrics (n=79)	5	0	0	61	13
	Physical medicine and rehabilitation (n=41)	4	1	0	30	6
	Plastic surgery-integrated (n=23)	0	3	0	18	2
	Psychiatry (n=52)	2	0	3	29	18
	Radiation oncology (n=24)	0	0	0	21	3
	Radiology-diagnostic (n=54)	1	0	0	52	1
	Surgery-general (n=71)	3	0	0	64	4
	Thoracic surgery-integrated (n=6)	0	1	0	5	0
	Urology (n=35)	3	4	0	27	1
	Vascular surgery-integrated (n=16)	1	0	0	14	1
	No specialty selected (n=110)	9	1	1	86	13
Total responses		75	37	10	981	171
Percentage of total responses		5.89%	2.90%	0.78%	77.00%	13.42%

## Discussion

This study found that 77.0% of the program directors that responded expect to continue utilizing an applicant's numerical USMLE Step 1 score as long as it's reported. This response was the most common across all medical specialties, with only family medicine and nuclear medicine being evenly split on the utility of numerical USMLE Step 1 scores during the 2023 application cycle.

The applicants who have taken USMLE Step 1 before January 26, 2022, have received a numerical score; our study found that the majority of program directors will continue to evaluate applicants similarly to previous application cycles. Thus, when selecting programs to apply to, applicants should consider the strength of their USMLE Step 1 score as central to their application.

The applicants who have taken USMLE Step 1 after January 26, 2022, will not receive a numerical score; these applicants can expect to be applying alongside applicants with numerical USMLE Step 1 scores that will be used during the residency selection process. How residency program directors will compare and contrast applicants with and without numerical scores has yet to be determined. Previous research done by Makhoul et al. in 2020 suggests that applicants without a numerical USMLE Step 1 score may begin to be evaluated based on their numerical USMLE Step 2 score [7].

In the coming years, there will continue to be residency applicants that present with a numerical USMLE Step 1 score. Particularly students who have taken a nontraditional undergraduate medical education course (i.e., students with a research year(s), concurrent programs such as MD/Ph.D., and students repeating clinical years). This study suggests that residency program directors will most likely continue to use numerical USMLE Step 1 scores for all applicants that have received one.

Some limitations of this study are the inherent challenge of survey studies in that some program directors may be more or less inclined to respond, resulting in the potential for nonresponse bias. Additionally, this survey was conducted shortly after the USMLE announced the change in scoring system, which has provided the opportunity for program directors to develop new systems to adjust to the challenges of evaluating applicants that do not all have a numerical USMLE Step 1 score.

## Conclusions

Our findings suggest that residency applicants with a numerical USMLE Step 1 score, independent of specialty, can expect that their score will likely be used during their 2023 residency selection process. Those residency applicants that did not receive a numerical USMLE Step 1 score present an active challenge for program directors to objectively compare the two cohorts.

## Figures and Tables

**Figure 1:**
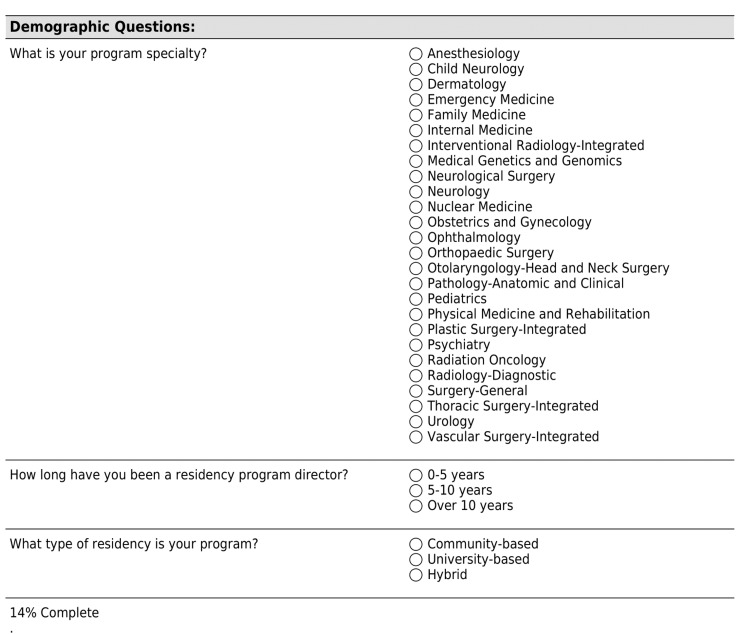
US MD residency directors survey - page 1.

**Figure 2:**
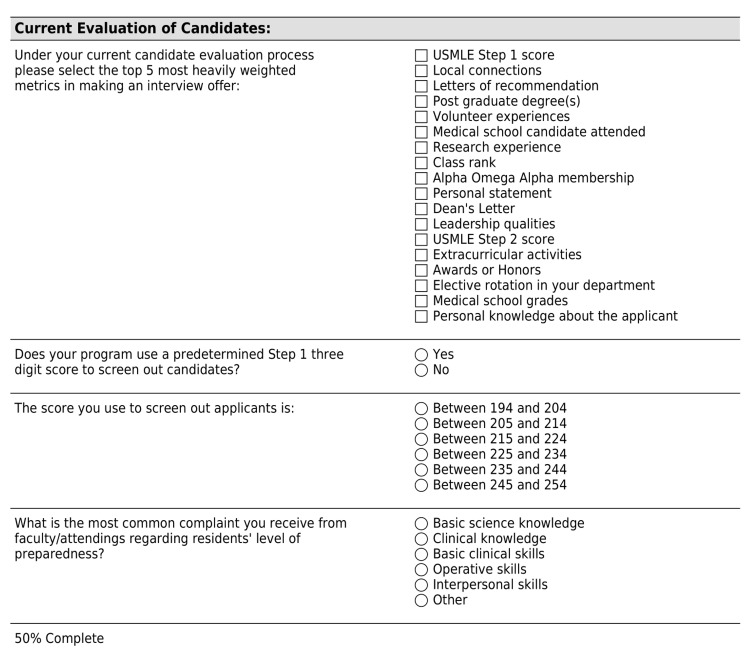
US MD residency directors survey - page 2.

**Figure 3:**
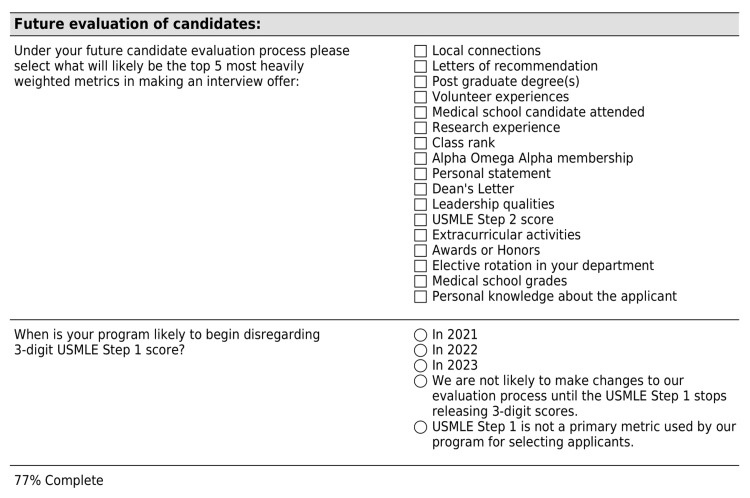
US MD residency directors survey - page 3.

**Figure 4:**
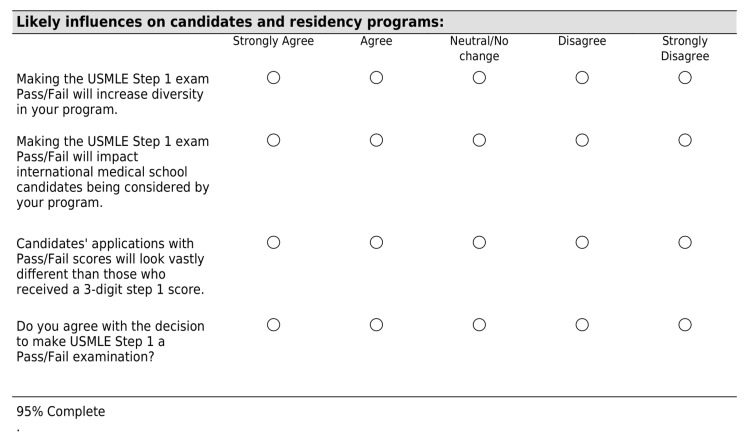
US MD residency directors survey - page 4.

**Figure 5:**
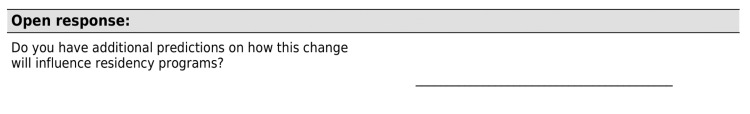
US MD residency directors survey - page 5.
